# Inclusion-Body Myopathy with Paget Disease of the Bone and Frontotemporal Dementia (IBMPFD) with *SCN4A* Mutation: Modifying Factor or Not?

**DOI:** 10.3390/diagnostics16172833

**Published:** 2026-09-03

**Authors:** Sekai Tsujimoto, Koji Hayashi, Mamiko Sato, Toshio Hamada, Asuka Suzuki, Yuka Nakaya, Toyoaki Miura, Ichizo Nishino, Wakako Yoshioka, Yasutaka Kobayashi

**Affiliations:** 1 Department of Rehabilitation Medicine, Fukui General Hospital, Fukui 910-8561, Japansatomoko@f-gh.jp (M.S.);; 2 Graduate School of Health Science, Fukui Health Science University, Fukui 910-3190, Japan; yasutaka_k@fukui-hsu.ac.jp; 3 Department of Neurology, Fukui Prefectural Hospital, Fukui 910-8526, Japan; 4 Department of Neuromuscular Research, National Institute of Neuroscience (NIN), National Center of Neurology and Psychiatry (NCNP), Tokyo 187-8502, Japan; nishino@ncnp.go.jp (I.N.); wyoshioka@ncnp.go.jp (W.Y.)

**Keywords:** myopathies/genetics, Paget disease of bone, frontotemporal dementia, valosin containing protein, NAV1.4 voltage-gated sodium channel, inclusion-body myopathy with early-onset Paget disease and frontotemporal dementia, osteitis deformans

## Abstract

A 56-year-old man with a family history of myopathy developed generalized muscle weakness at age 45. At age 52, he was diagnosed with inclusion-body myopathy with Paget disease of bone and frontotemporal dementia (IBMPFD), confirmed by muscle pathology and a pathogenic *VCP* mutation (c.464G>A, p.R155H). Concurrent testing identified a heterozygous *SCN4A* variant (c.215C>T, p.P72L), a known modifier in myotonic dystrophy type 2 (DM2). At age 56, neurological examination showed proximal muscle weakness (MMT grade 3), distal lower extremity weakness (grade 4–5), and areflexia. He ambulated independently with knee locking, though squatting was impossible. No myotonia or periodic paralysis was observed. Multimodal imaging captured classic IBMPFD hallmarks: skeletal muscle computed tomography (CT) showed advanced fatty degeneration of paraspinal and limb muscles; brain magnetic resonance imaging (MRI) revealed mild frontal lobe atrophy; and bone scintigraphy showed increased uptake in the lumbar spine and iliums. Although the *SCN4A* p.P72L variant exacerbates DM2, the patient’s three-year progression from independent walking to cane use remained consistent with the natural history of IBMPFD. This case suggests that in the presence of a robustly penetrant *VCP* phenotype, the *SCN4A* variant does not necessarily exert a clinical modifying effect.

**Figure 1:**
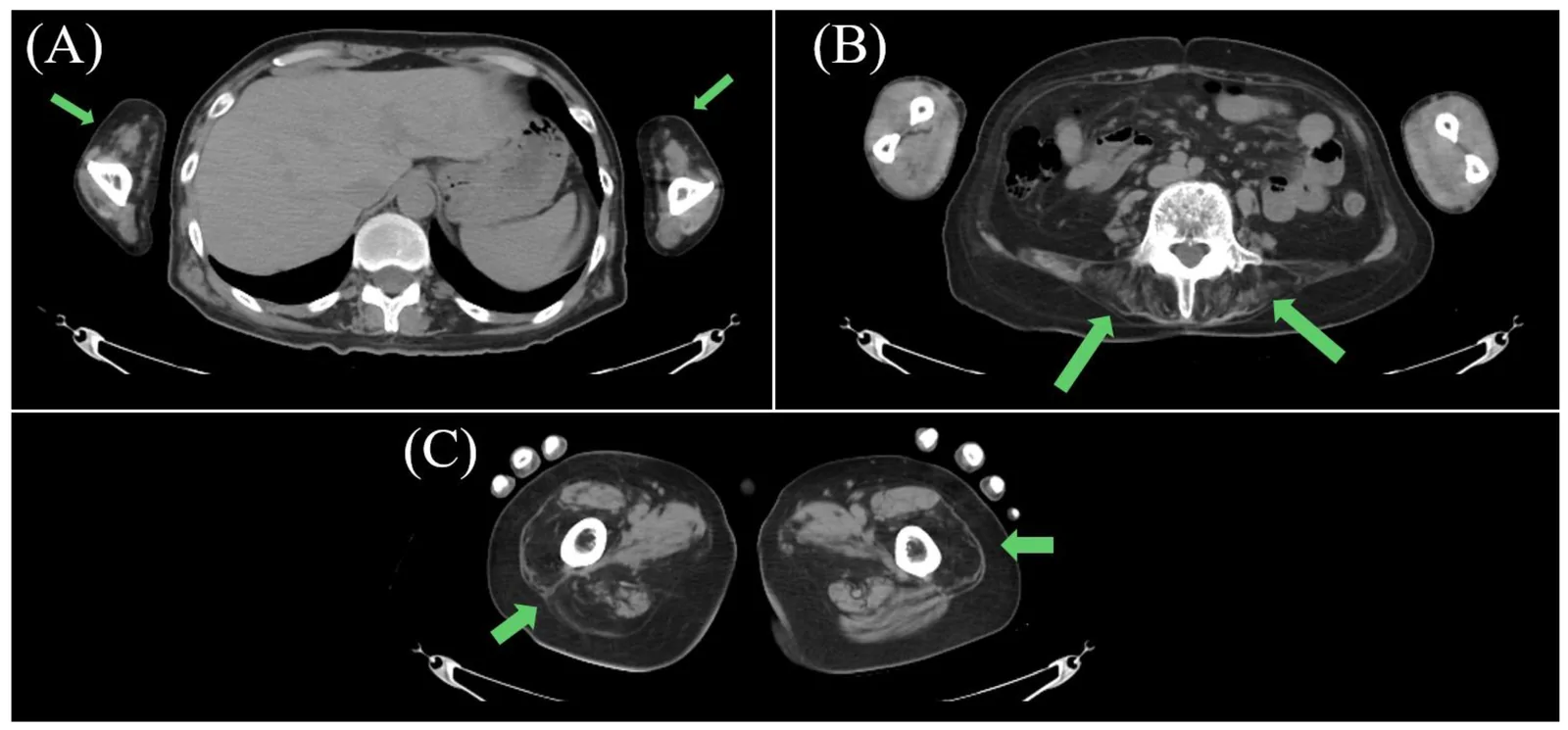
Muscle computed tomography (CT) findings. The CT scan showing muscle atrophy predominantly in the proximal muscles, including the upper arms (**A**), paraspinal muscles (**B**), and thighs (**C**) (green arrows).

**Figure 2:**
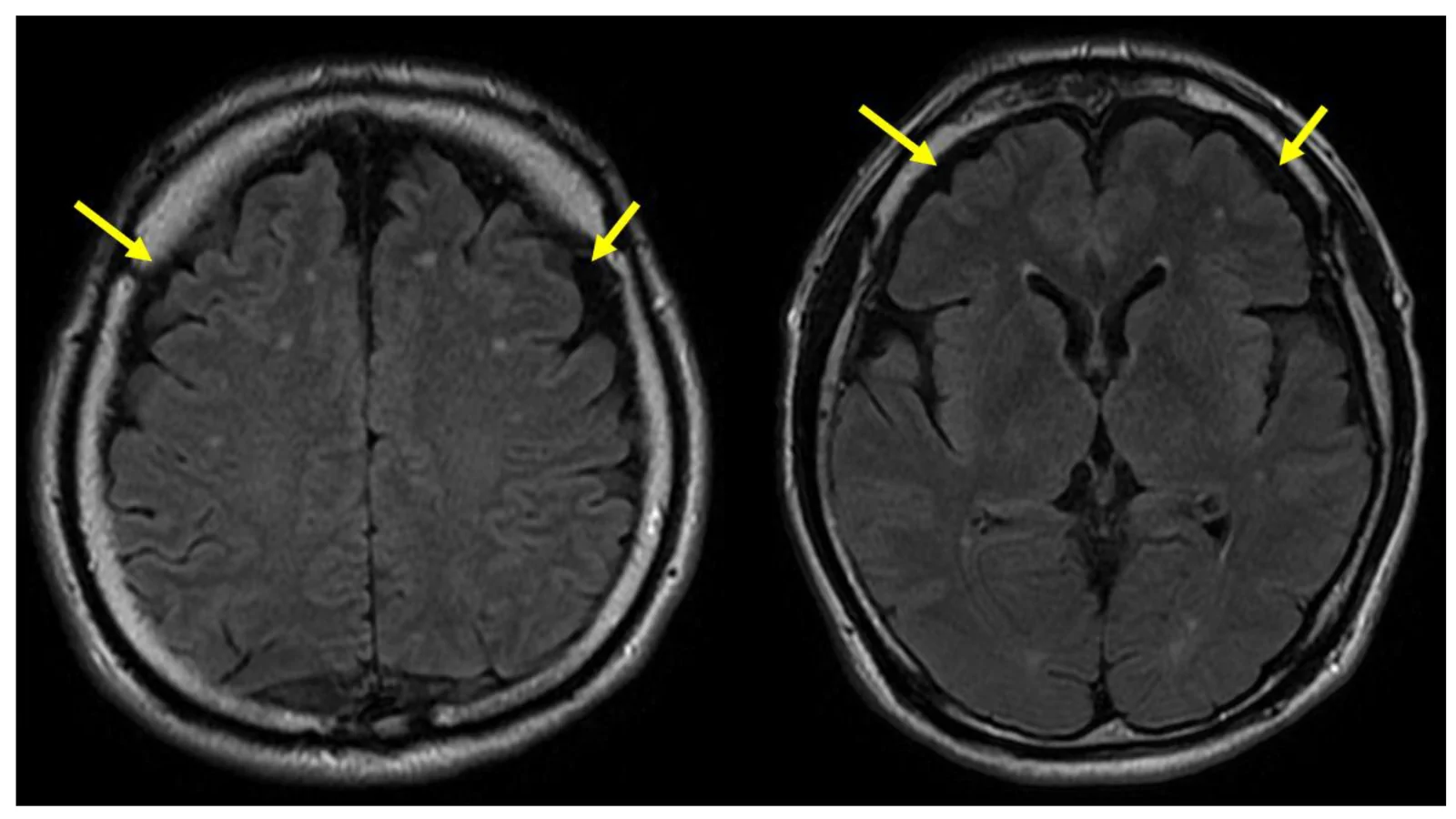
Brain magnetic resonance imaging (MRI). T2-weighted fluid-attenuated inversion recovery (FLAIR) images showing mild atrophy in the bilateral frontal lobes (yellow arrows).

**Figure 3:**
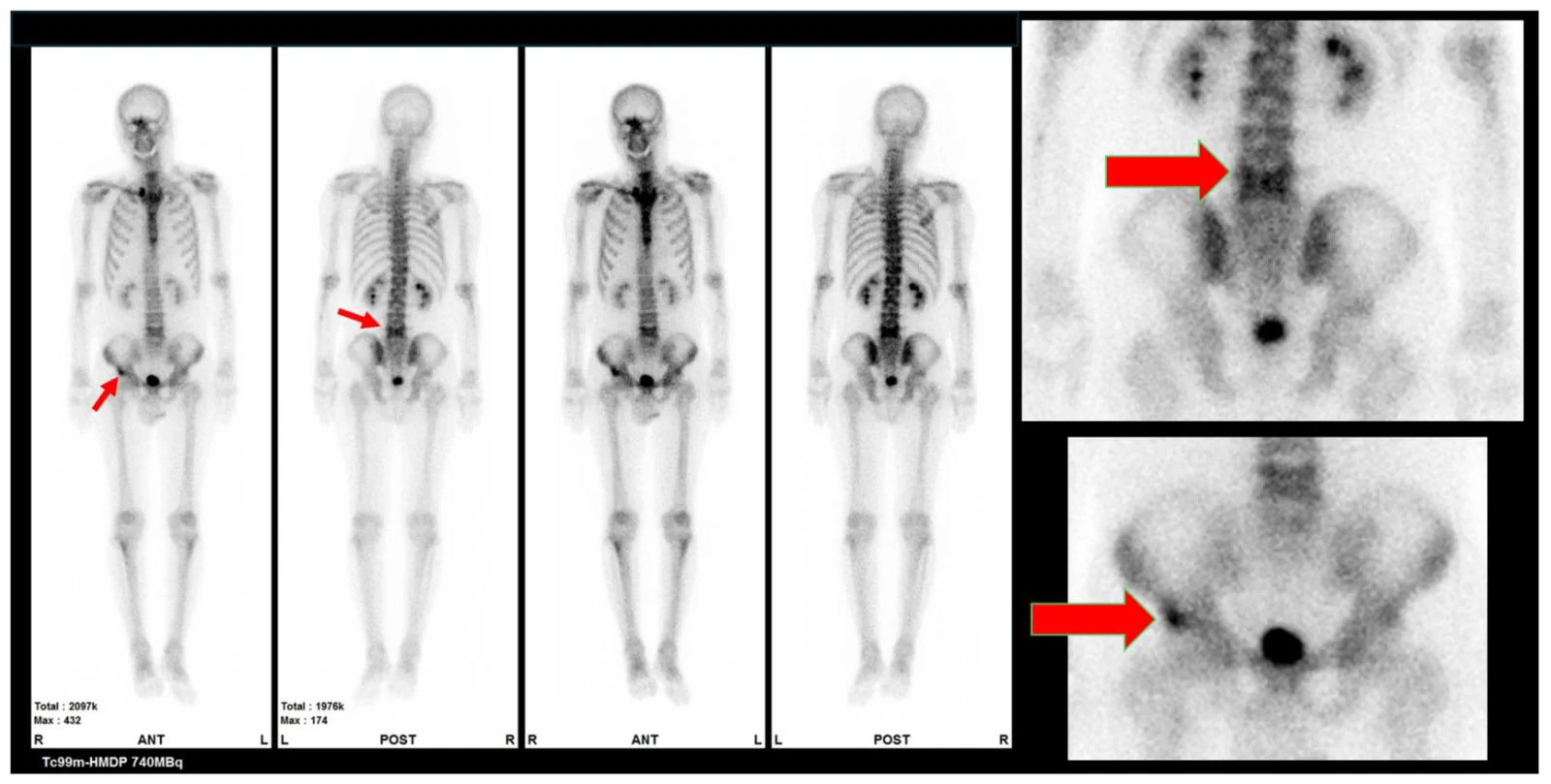
^99m^Tc-HMDP bone scintigraphy. Increased uptake suggestive of Paget disease of bone is observed in the pelvis and vertebral bodies (red arrows).

**Figure 4:**
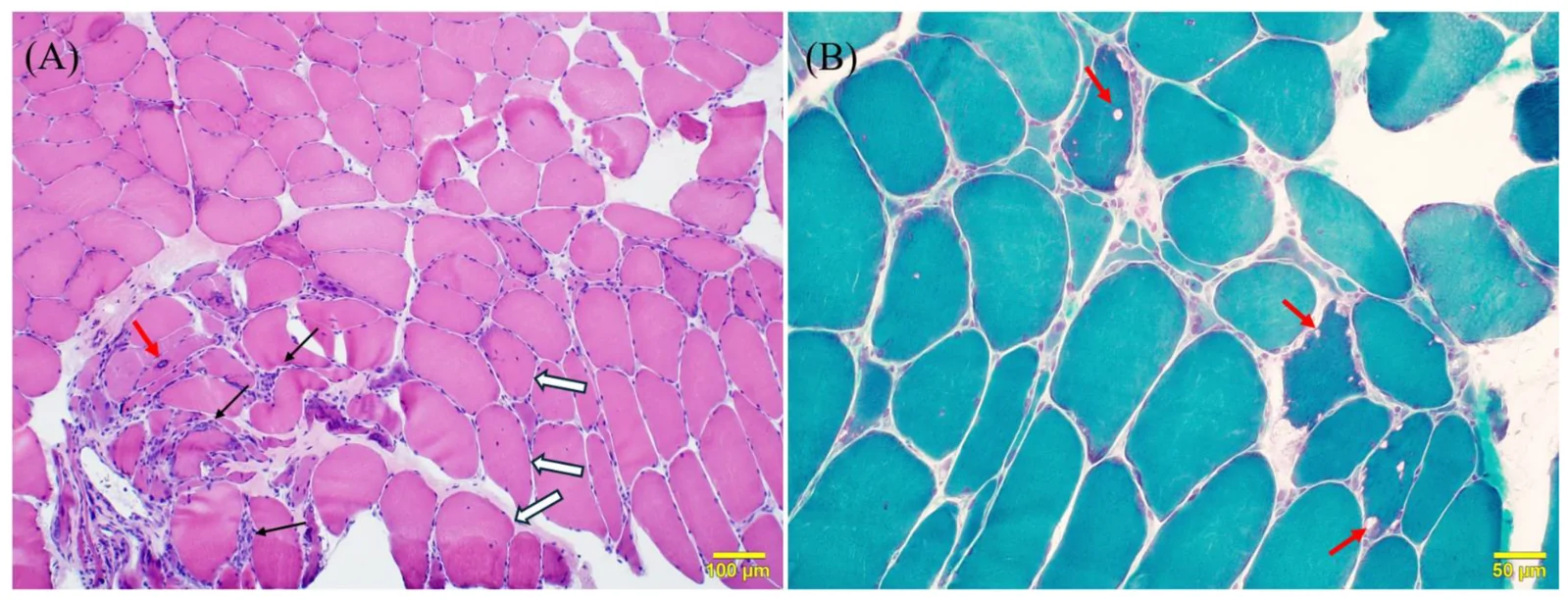
Muscle biopsy findings. (**A**) On hematoxylin and eosin (H&E) staining, mild variation in muscle fiber size is observed, together with endomysial fibrosis (black arrows), fibers with centrally located nuclei (white arrows), and fibers containing vacuoles (red arrows). (**B**) On modified Gomori trichrome staining, fibers with rimmed vacuoles are noted (red arrows). The histopathological findings in Figure 4, including variation in muscle fiber size and internalized nuclei, reflect a chronic myopathic process [1,2]. The hallmark feature is the presence of rimmed vacuoles (RVs), which represent a fundamental failure in autophagosome maturation [3,4]. Unlike sporadic inclusion-body myositis (sIBM), this case lacks significant inflammatory infiltration, a key diagnostic separator for *VCP*-associated disease [1,2].

**Figure 5:**
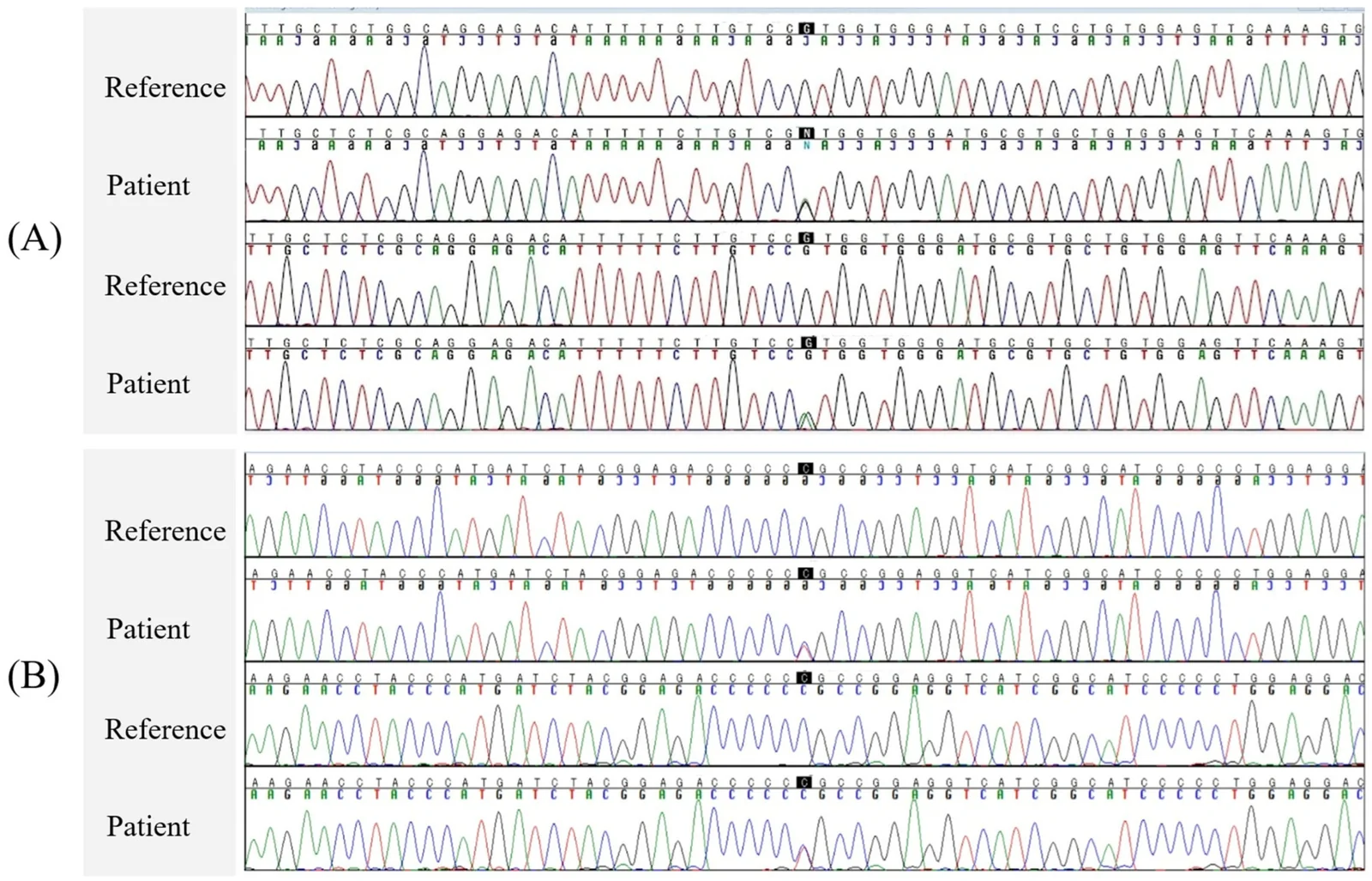
Results of Sanger sequencing. (**A**) Analysis of the *VCP* gene revealed a heterozygous c.464G>A variant in exon 5 (NM_007126.5), which results in a p.R155H amino acid substitution. This p.R155H variant is a well-established pathogenic variant strongly associated with *VCP*-associated multisystem proteinopathy, including inclusion-body myopathy with Paget disease of the bone and frontotemporal dementia (IBMPFD) or the scapuloperoneal syndrome [1,4,5,6,7,8,9,10,11,12,13]. (**B**) Analysis of the *SCN4A* gene identified a heterozygous c.215C>T (p.P72L) variant in exon 1 (NM_000334.4). According to gnomAD [14], this variant is extremely rare (allele frequency: 0.006%) and is currently classified as a variant of uncertain significance (VUS) based on the American College of Medical Genetics and Genomics (ACMG) criteria [15]. Although the amino acid substitution preserves the nonpolar property (proline to leucine), it eliminates the structural constraint unique to proline residues, potentially affecting local protein folding. Functional studies of the *SCN4A* p.P72L variant demonstrated a hyperpolarizing shift in the voltage dependence of channel activation, suggesting increased membrane excitability [16]. Although the role of *SCN4A* variants as clinical modifiers has been established in myotonic dystrophy type 2 (DM2) by enhancing membrane hyperexcitability, their impact on other neuromuscular disorders remains to be elucidated [16,17]. Crucially, the modifier effect of *SCN4A* in DM2 relies on an additive interaction with an underlying channelopathy background (i.e., CLC-1 dysfunction) [17]. In contrast, IBMPFD is fundamentally a degenerative proteinopathy driven by impaired protein homeostasis and autophagy, lacking a primary ion channel defect [1,2,3,5,7,11,18,19]. Therefore, the increased electrical excitability conferred by the *SCN4A* variant is unlikely to interact with or accelerate the proteotoxic and vacuolar degenerative pathways characteristic of *VCP* disease. In our patient, despite the presence of the *SCN4A* p.P72L variant, the clinical phenotype was entirely representative of classic IBMPFD, with no additional myotonic or atypical features. This suggests that in the context of *VCP*-associated disease, this *SCN4A* variant represents an incidental co-occurrence rather than a substantial clinical modifier. Ultimately, this case underscores the indispensable role of comprehensive clinical and radiological phenotyping in interpreting complex genetic findings in the genomic era. Further studies are warranted to accumulate and evaluate the potential modifying effects of *SCN4A* variants across a broader range of non-myotonic muscle diseases.

## Data Availability

The data presented in this study are available on request from the corresponding author. Due to patient privacy and ethical considerations, the data are not publicly accessible.

## References

[1] Schiava M., Parkhurst Y., Henderson M., Polvikoski T., Valtcheva M.V., Nishino I., Inoue M., Nishimori Y., Saito Y., Stojkovic T. (2025). Muscle biopsy findings in valosin-containing protein multisystem proteinopathy. Neurol. Genet..

[2] Tresse E., Salomons F.A., Vesa J., Bott L.C., Kimonis V., Yao T.-P., Dantuma N.P., Taylor J.P. (2010). VCP/p97 is essential for maturation of ubiquitin-containing autophagosomes and this function is impaired by mutations that cause IBMPFD. Autophagy.

[3] Ju J.S., Weihl C.C. (2010). p97/VCP at the intersection of the autophagy and the ubiquitin proteasome system. Autophagy.

[4] Jerath N.U. (2019). Resolving a multi-generational neuromuscular mystery in a family presenting with a variable scapuloperoneal syndrome in a c.464G>A, p.Arg155His VCP mutation. Case Rep. Genet..

[5] Iannibelli E., Gibertini S., Cheli M., Blasevich F., Cavaliere A., Riolo G., Ruggieri A., Maggi L. (2023). VCP-related myopathy: A case series and a review of literature. Acta Myol..

[6] Columbres R.A., Chin Y., Pratti S., Quinn C., Gonzalez-Cuyar L.F., Weiss M., Quintero-Rivera F., Kimonis V. (2023). Novel Variants in the *VCP* Gene Causing Multisystem Proteinopathy 1. Genes.

[7] Ferrari V., Cristofani R., Tedesco B., Crippa V., Chierichetti M., Casarotto E., Cozzi M., Mina F., Piccolella M., Galbiati M. (2022). Valosin Containing Protein (VCP): A Multistep Regulator of Autophagy. Int. J. Mol. Sci..

[8] Shmara A., Perez-Rosendahl M., Murphy K., Kwon A., Smith C., Kimonis V. (2022). A clinicopathologic study of malignancy in VCP-associated multisystem proteinopathy. Orphanet J. Rare Dis..

[9] Chan J.M., Romano C., Lee A.Y., Wang S., Lombardo D., Kamdar F., Dora M., Khan S., Mammen P., Kimonis V. (2025). Cardiomyopathy in valosin-containing protein multisystem proteinopathy: Evaluation, diagnosis, and management. Am. Heart J. Plus.

[10] Wan Y., Wang Q., Zheng Y., Yu M., Xie Z., Ling C., Meng L., Yu J., Zheng Y., Wang Y. (2023). Novel variants, muscle imaging, and myopathological changes in Chinese patients with VCP-related multisystem proteinopathy. Mol. Genet. Genom. Med..

[11] Luzzi A., Wang F., Li S., Iacovino M., Chou T.F. (2023). Skeletal muscle cell protein dysregulation highlights the pathogenesis mechanism of myopathy-associated p97/VCP R155H mutations. Front. Neurol..

[12] Pfeffer G., Lee G., Pontifex C.S., Fanganiello R.D., Peck A., Weihl C.C., Kimonis V. (2022). Multisystem proteinopathy due to VCP mutations: A review of clinical heterogeneity and genetic diagnosis. Genes.

[13] Takata T., Kimura Y., Ohnuma Y., Kawawaki J., Kakiyama Y., Tanaka K., Kakizuka A. (2012). Rescue of growth defects of yeast cdc48 mutants by pathogenic IBMPFD-VCPs. J. Struct. Biol..

[14] Karczewski K.J., Francioli L.C., Tiao G., Cummings B.B., Alfoldi J., Wang Q., Collins R.L., Laricchia K.M., Ganna A., Birnbaum D.P. (2020). The mutational constraint spectrum quantified from variation in 141,456 humans. Nature.

[15] Richards S., Aziz N., Bale S., Bick D., Das S., Gastier-Foster J., Grody W.W., Hegde M., Lyon E., Spector E. (2015). Standards and guidelines for the interpretation of sequence variants: A joint consensus recommendation of the American College of Medical Genetics and Genomics and the Association for Molecular Pathology. Genet Med..

[16] Binda A., Renna L.V., Bosè F., Brigonzi E., Botta A., Valaperta R., Fossati B., Rivolta I., Meola G., Cardani R. (2018). SCN4A as modifier gene in patients with myotonic dystrophy type 2. Sci. Rep..

[17] Bugiardini E., Rivolta I., Binda A., Caminero A.S., Cirillo F., Cinti A., Giovannoni R., Botta A., Cardani R., Wicklund M. (2015). SCN4A mutation as modifying factor of myotonic dystrophy type 2 phenotype. Neuromuscul. Disord..

[18] Chae J., Park S.H., Sung J.J. (2024). VCP-related multisystem proteinopathy presenting with lobulated myofibers. Korean J. Neuromuscul. Disord..

[19] Jang H., Jang E.R., Wilson P.G., Anderson D., Galperin E. (2019). VCP/p97 controls signals of the ERK1/2 pathway transmitted via the Shoc2 scaffolding complex: Novel insights into IBMPFD pathology. Mol. Biol. Cell.

